# Short-Acting Beta-Agonists, Antibiotics, Oral Corticosteroids, and the Associated Burden of COPD

**DOI:** 10.1016/j.chpulm.2024.100042

**Published:** 2024-02-19

**Authors:** Mohit Bhutani, Arsh Randhawa, Manisha Talukdar, Phongsack Manivong, Danielle Fearon, Aaron Gelfand, Erin Graves, Suzanne McMullen, Irvin Mayers

**Affiliations:** a University of Alberta, Edmonton, AB, Canada; b Medlior Health Outcomes Research Ltd., Calgary, AB; c AstraZeneca, Mississauga, ON, Canada

**Keywords:** COPD, disease burden, drug therapy, exacerbation

## Abstract

**Background:**

Severe acute exacerbations of COPD (AECOPDs) are key events that drive health care resource use (HCRU) and negatively impact patients’ quality of life.

**Research Question:**

What is the real-world burden of COPD relative to patients’ medication history, specifically, exposure to short-acting beta-agonists (SABAs), antibiotics, and oral corticosteroids (OCSs)?

**Study Design and Methods:**

A population-based retrospective cohort study was conducted of patients in Alberta, Canada, identified as having COPD based on administrative health care data (April 1, 2011-March 31, 2019). The risk of severe AECOPDs over 90 days (COPD events resulting in hospitalization or ED visits) and COPD-specific HCRU were studied relative to prior-year SABA, antibiotic, and OCS history.

**Results:**

One hundred eighty-eight thousand nine hundred sixty-nine patients identified with COPD were identified (mean ± SD age, 68.8 ± 13.0 years). After controlling for age, sex, calendar year at index, comorbidities at index, and prior severe AECOPDs, patients with frequent SABA, antibiotic, or OCS exposure in a given year showed significantly higher 90-day risks of severe AECOPDs in a positively associated relationship. Patients with the highest SABA exposure (≥ 6 canisters in a given year) showed twice the rate of severe AECOPDs as patients with 1 SABA canister (incidence rate ratio [IRR], 2.06; 95% CI, 2.01-2.11). The 90-day rates of severe AECOPDs were 51% higher for patients with ≥ 6 vs 1 to 2 antibiotic dispensations (IRR, 1.51; 95% CI, 1.48-1.55) and 3% higher for patients with ≥ 6 vs 1 to 5 OCS burst days (IRR, 1.03; 95% CI, 1.00-1.06). Mean annualized rates of hospitalization and ED visits were highest for patients dispensed ≥ 6 (vs fewer) SABA canisters or antibiotics and patients with any OCS burst days in a given year.

**Interpretation:**

Histories of frequent or prolonged exposure to SABAs, antibiotics, or OCSs were associated with higher rates of severe AECOPDs and HCRU.

**Take-home Points**

**Study Question:** What is the real-world burden of COPD relative to patients’ medication history, specifically short-acting beta-agonists (SABAs), antibiotics, and oral corticosteroids (OCSs)?

**Results:** Patients with frequent SABA, antibiotic, or OCS dispensation in a given year showed higher 90-day rates of severe acute exacerbations in COPD, as well as generally higher annualized rates of hospitalizations, ED visits, general practitioner or specialist visits, and direct health care costs.

**Interpretation:** Medication history may be a proxy to identify and assess the risk of future severe exacerbations, health care resource use, and costs.

COPD is a leading cause of death worldwide, responsible for an estimated 3.3 million deaths annually.^1^ In the fiscal year 2011 to 2012, the estimated age-standardized prevalence of COPD was 9% for Canadians aged ≥ 35 years.^2^ Additionally in Canada from 2016 through 2021, COPD and bronchitis were the second-leading causes of hospitalization.^3^ Therefore, COPD represents a considerable burden on patients and health care systems,^4^ contributing to higher health care costs in Canada^5^ and worldwide.^6^

Acute exacerbations of COPD (AECOPDs) are key events that drive health care resource use (HCRU)^7^ and negatively impact patients’ quality of life.^8^ A shared goal of clinicians and researchers is to identify patients at higher risk of exacerbations as early as possible, offering greater opportunity to prevent future exacerbations.

Pharmacologic treatments in COPD should be individualized and targeted to reduce symptoms, to improve quality of life and clinical outcomes, and to reduce the frequency and severity of exacerbations.^9^ The 2019 Canadian Thoracic Society COPD pharmacotherapy guidelines advise patients at high risk of exacerbations to start with long-acting muscarinic antagonists and long-acting beta-2 agonists or inhaled corticosteroids and long-acting beta-2 agonists and to step up to triple therapy as maintenance medications.^9^ To address AECOPDs and increased symptoms, additional agents typically are administered, including short-term oral corticosteroids (OCSs), antibiotics, and rescue medications, such as short-acting beta-agonists (SABAs) or short-acting muscarinic antagonist.^10^ Assessing the relationship between medication history and the occurrence of severe AECOPDs may help clinicians and researchers to identify patients at risk of worse outcomes.

The purpose of this study was to identify opportunities for improved treatment and management of patients living with COPD to improve their prognosis. More specifically, we aimed to understand how SABA, antibiotic, and OCS dispensation history could be used as predictors or as proxies to identify patients at risk. Thus, the study objectives were to estimate the 90-day risks of severe AECOPDs and the annualized COPD-specific HCRU and direct costs, based on 1-year history of SABA (primary objective), antibiotic, and OCS (secondary objectives) use.

## Study Design and Methods

### Study Design and Data Sources

This was a retrospective population-based cohort study of patients identified as having COPD in Alberta, Canada. Population-based administrative data were acquired from Alberta Health (Supplementary Material 1).

### Case Definitions

The COPD cohort was derived from individuals identified in Alberta Health’s COPD chronic disease cohort^11^ between April 1, 1985, and March 31, 2021. Patients were identified as having COPD by using an accepted definition^12^ with a positive predictive value of 72.6% (95% CI, 62.8-80.9) and the following criteria: ≥ 1 International Classification of Diseases (ICD), 10th Revision, Canada (hospitalization), diagnosis code for COPD (J41-J44) in any position or ≥ 2 ICD, Ninth Revision, Clinical Modification (physician visits), diagnosis codes for COPD (491, 492, and 496) in the primary position within a 2-year period at baseline.

The main analytical cohort for this study showed a case ascertainment period of April 1, 2011, through March 31, 2019, and maximum follow-up ending on March 31, 2020. This was to capture patients with at least 1 year of potential follow-up before the COVID-19 pandemic. Eligible patients were required to be aged ≥ 35 years at index and to reside in Alberta for at least 1 year before and after index to ensure patients have linkable data during follow-up. For incident cases, the index date was the COPD diagnosis date. Prevalent patients were those who received a diagnosis of COPD before April 1, 2011, and were assigned an index date of April 1, 2011. Patients were followed up until study end, date of death, or discontinuation of registration in Alberta, whichever occurred first.

### Variables

Demographic and clinical variables included: age (years) at index date, sex, the event leading to identification in the cohort (hospitalization or physician visit), COPD-related prescription dispensations 1 year before index date, and Charlson Comorbidity Index (CCI). The CCI was derived using methods from Quan et al^13^ based on inpatient hospitalizations 1 year before and including the index date.

**Figure 1:**
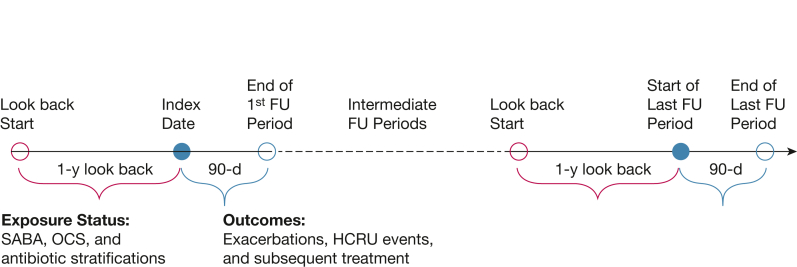
Diagram showing exposures and outcomes, analytic approach. For each FU period, a 1-year look-back window was used to determine prior medication history. FU time and outcomes were counted only once in the respective FU period. Activity in each 1-year look-back window may have contributed to exposure status in multiple FU periods. FU = follow-up; HCRU = health care resource use; OCS = oral corticosteroid; SABA = short-acting beta-agonist.

To allow for time-varying risk, cohort follow-up was divided into periods of up to 90 days, starting from the index date and finishing at the end of follow-up. COPD medication exposure was assessed for SABAs, antibiotics, and OCSs in the year before each follow-up period. Therefore, we periodically assessed the frequency of COPD medication dispensation throughout follow-up, rather than assigning an average or single level of exposure. We also assessed the rate of severe AECOPDs at the start of each follow-up. Outcomes were assessed during each 90-day interval (Fig 1). This was a modest approach to capture changes in risk throughout follow-up that were frequent but not instantaneous that allowed us to assess the temporal association between medications and exacerbations.

SABA dispensations were defined as the number of canisters dispensed (Supplementary Material 2). Antibiotic use was reported as the number of dispensations. OCS burst days were the number of days of an OCS burst, defined as an OCS prescription (of at least 3-28 days) for an average daily dose of ≥ 20 mg prednisone combined with a physician or ED visit within 7 days before or after the pharmacy dispensation. Stratification categories then were created for these COPD medications based on the 1-year lookback use history. The initial category stratifications for these medications were derived first by assessing the distribution of non-zero values and then by grouping dispensation values based on the statistical significance of associations with severe AECOPDs. This was an iterative approach that resulted in a practical number of medication-exposure categories (strata) for analysis and to facilitate clinical interpretability. Because our main research interest was among medication users, the first (lowest) non-zero category was used as the reference (1 SABA canister dispensation, 1-2 antibiotic dispensations, or 1-5 OCS burst days).

Outcome variables were severe AECOPDs (defined as an ED visit or hospitalization with COPD as the primary ICD, 10th Revision, Canada, diagnosis) and HCRU (including hospitalizations, ED visits, physician encounters, length of stay in hospital [days] or ED [h], and COPD medication dispensations). Severe AECOPDs within 14 days of each other were grouped into a single exacerbation event.

COPD-specific HCRU was identified by the presence of an ICD, Ninth Revision, Clinical Modification, code for COPD for physician visits and a COPD ICD, 10th Revision, Canada, primary diagnosis code for ambulatory and hospitalization HCRU. Medication regimen categories were derived using methods shown in Supplementary Material 3. Direct costs were estimated using methods shown in Supplementary Material 4.

### Statistical Analysis

Patient characteristics at index were summarized descriptively for the total cohort. Associations between severe AECOPDs within 90-day follow-up periods and 1-year prior history of SABA treatment, antibiotic treatment, and OCS burst days were modelled using Poisson regression adjusted for age, sex, CCI at index, year of index, and 1-year prior severe AECOPDs as control variables. To account for repeat observations for the same individual over time, SEs were calculated using generalized estimating equation methodology.

Three post hoc sensitivity analyses were performed. Acknowledging that OCS burst days and 1-year prior exacerbations were collinear (because both included ED visits), the first sensitivity analysis removed prior exacerbations from the risk-adjusted model to reassess the effect of OCS burst days. To account for maintenance treatment, the second included dispensation of long-acting beta-2 agonists, long-acting muscarinic antagonists, or both in the year before each follow-up period. The third accounted for the 1-year prior history of inpatient hospitalization, ED visits, and practitioner visits.

HCRU and costs were stratified by SABA and antibiotic dispensations and OCS burst days in the 1 year preceding each follow-up period (Fig 1). Descriptive statistics were used to characterize the occurrence and frequency of COPD-specific HCRU events. For each medication stratum, annualized event rates (eg, of hospitalization) were calculated as the sum of events divided by the number of follow-up years using only follow-up periods with the relevant medication category, per person. Cost calculations that included patients with no HCRU activity were assigned zero costs. Finally, the number and proportion of patients receiving medication regimens during follow-up were calculated.

All statistical analyses were performed using SAS version 9.4 software (SAS Institute). Where appropriate, statistical significance was identified from *P* < .05 or when the incidence rate ratio (IRR) 95% CI did not contain the value of 1. This study was approved by the Health Research Ethics Board of Alberta-Community Health Committee (Identifier, HREBA.CHC-21-0011). The authors used the Strengthening the Reporting of Observational Studies in Epidemiology guidelines for writing this report.^14^

## Results

The study cohort included 188,969 patients (Supplementary Material 5), including prevalent and incident cases (Supplementary Material 6 provides annual counts over time). The mean ± SD and median (interquartile range [IQR]) follow-up times were 4.9 ± 3.2 years and 4.7 years (IQR, 2.0-8.6 years), respectively, for the total cohort.

Table 1 provides baseline characteristics for all patients with COPD in the study. Additional baseline characteristics are in Supplementary Material 7. The mean patient age ± SD at index was 68.8 ± 13.0 years; 52.1% of patients were male. Nearly one-quarter of patients (24.7%) met the COPD diagnosis criteria at hospitalization (see Case Definitions). Most patients (75.9%) did not undergo hospitalizations in the year before the index date to contribute to CCI. More than one-half of patients (54.5%) were dispensed some COPD-related medication in the year before the index date (Table 1).

**Table 1:** Demographic and Clinical Characteristics at Index Date for Patients With COPD in Alberta, Canada, April 2011-March 2019^a^

Characteristic	Overall
Total	
No. of patients	188,969 (100.0)
Age, y	
Mean ± SD	68.8 ± 13.0
Median (IQR)	69.0 (59.0-79.0)
35-49	12,969 (6.9)
50-64	60,383 (32.0)
65-79	71,560 (37.9)
≥ 80	44,057 (23.3)
Sex	
Female	90,485 (47.9)
Male	98,484 (52.1)
CCI comorbidity^b^	
Myocardial infarction	4,314 (2.3)
Congestive heart failure	7,670 (4.1)
Peripheral vascular disease	2,314 (1.2)
Diabetes without chronic complications	6,379 (3.4)
Diabetes with chronic complications	7,649 (4.0)
CCI (weighted score) categoric^b^	
0	22,134 (11.7)
1	6,177 (3.3)
2	8,577 (4.5)
3+	8,668 (4.6)
0, hospitalization within the defined time window for CCI	143,413 (75.9)
Event leading to identification	
Hospitalization	46,736 (24.7)
Physician visits	142,233 (75.3)
COPD-related prescription dispensations in any combination regimen 1 y before index date^c^	
Any COPD medication	102,912 (54.5)
OCS	36,625 (19.4)
SABA	65,482 (34.7)
SAMA	13,537 (7.2)
Antibiotic	104,642 (55.4)
PDE4 inhibitor	30 (0.0)
ICS	68,598 (36.3)
LAMA	43,635 (23.1)
LABA	59,731 (31.6)

Data are presented as No. (%), unless otherwise indicated. CCI = Charlson Comorbidity Index; DAD = Discharge abstract database; ICS = inhaled corticosteroid; IQR = interquartile range; LABA = long-acting beta-2 agonist; LAMA = long-acting muscarinic antagonist; OCS = oral corticosteroid; PDE4 = phosphodiesterase 4; SABA = short-acting beta-agonist; SAMA = short-acting muscarinic-antagonist.

a COPD International Classification of Diseases, 10th Revision, Canada, codes for cohort: J41, J42, J43, and J44. Chronic pulmonary disease International Classification of Diseases, 10th Revision, Canada, codes in the CCI included: I27.8, I27.9, J40.x-J47.x, J60.x-J67.x, J68.4, J70.1, and J70.3.

b Individuals were classified as having CCI comorbidities based on diagnoses recorded within 1 year before index date based on hospital abstracts (DAD) only.

c COPD medication history before index based on anatomic therapeutic chemical classification codes (combination therapy not included).

The unadjusted model (Table 2) showed a positively associated effect between 90-day risk of severe AECOPD and 1-year history of SABA and antibiotic dispensations and OCS burst days, where the rates of severe AECOPDs were higher with more frequent medication dispensations. Notably, at the highest risk level, patients with ≥ 6 SABA dispensations (vs 1) showed over twice the rate of severe AECOPDs (risk-adjusted IRR, 2.06; 95% CI, 2.01-2.11). Similarly, at the highest risk level, patients dispensed ≥ 6 antibiotics (vs 1-2) in a given year showed a 51% higher rate of exacerbations (risk-adjusted IRR, 1.51; 95% CI, 1.48-1.55). Patients with ≥ 6 OCS burst days (vs 1-5) in a given year showed a 3% higher rate of subsequent exacerbations, which was not significant (risk-adjusted IRR, 1.03; 95% CI, 1.00-1.06). When the history of exacerbations in the prior year was removed from the model in a sensitivity analysis, this rate of subsequent exacerbations became 32% higher and statistically significant (IRR, 1.32; 95% CI, 1.28-1.36) (Supplementary Material 8). The other sensitivity analyses yielded similar results to the main analysis.

**Table 2:** Associations Between 90-Day Severe Exacerbations and 1-Year Prior History of Medication Dispensation in Patients With COPD in Alberta, Canada, April 2011-March 2020 (n = 188,969)^a^

Medication Dispensed (Units)	1-Y Medication History^b^	Unadjusted			Risk Adjusted^c^		
		IRR	95% CI	*P* Value	IRR	95% CI	*P* Value
SABAs (canisters)	0	0.45	0.44-0.46	< .001	0.56	0.55-0.57	< .001
	1	1.00	Reference	...	1.00	Reference	...
	2-5	1.48	1.44-1.51	< .001	1.35	1.33-1.38	< .001
	6+	2.71	2.64-2.79	< .001	2.06	2.01-2.11	< .001
Antibiotics (dispensations)	0	0.54	0.53-0.55	< .001	0.73	0.72-0.75	< .001
	1-2	1.00	Ref	...	1.00	Reference	...
	3-5	1.74	1.71-1.77	< .001	1.28	1.25-1.30	< .001
	6+	2.91	2.82-2.99	< .001	1.51	1.48-1.55	< .001
OCSs (burst days)	0	0.20	0.19-0.20	< .001	0.46	0.45-0.47	< .001
	1-5	1.00	Reference	...	1.00	Reference	...
	6+	1.62	1.57-1.68	< .001	1.03	1.00-1.06	.053

IRR = incidence rate ratio; OCS = oral corticosteroid; SABA = short-acting beta-agonist.

a IRRs were modelled using a Poisson regression with severe exacerbations as the outcome variable.

b Medication history categories were defined using empirically derived thresholds and were based on the 1-y history.

c Risk-adjusted model included: SABA dispensations (1-y history), antibiotic dispensations (1-y history), OCS burst days (1-y history), sex, age in years at index date, calendar year of index date, 1-y prior severe COPD exacerbation rate, and Charlson comorbidity index at index.

Patients with a history of ≥ 6 SABA dispensations, ≥ 6 antibiotic dispensations, or 1 to 5 OCS burst days in any given year showed the highest annualized rates of hospitalizations, ED visits, and general practitioner or specialist visits in the subsequent 90 days of follow-up, often with a positively associated effect, compared with all other treatment profiles (Table 3). Notably, the annualized rates of ED visits for patients with ≥ 6 SABA dispensations or ≥ 6 antibiotic dispensations were more than twice the rates for patients with 1 SABA dispensation or 1 to 2 antibiotic dispensations, respectively (SABA, mean ± SD, 0.5 ± 3.0 vs 0.2 ± 2.5; antibiotics, mean ± SD, 0.4 ± 4.2 vs 0.2 ± 2.9).

**Table 3:** Annualized COPD-Specific HCRU by 1-Year Prior History of Medication Dispensation for Patients in Alberta, Canada, April 2011-March 2020

Variable	All Patients	1-Y Medication History Before 90-D Follow-up Intervals										
		SABA Canisters^a^				Antibiotic Dispensations^a^				OCS Burst Days^a^		
		0	1	2-5	6+	0	1-2	3-5	6+	0	1-5	6+
Patients contributing to follow-up time^c^	188,969	158,327	87,608	83,526	45,659	154,046	154,552	96,608	38,042	188,091	16,469	16,249
Follow-up per patient, person-y	4.7 (2.0-8.6)	3.0 (1.0-6.0)	1.0 (0.4-1.5)	1.2 (0.6-2.2)	1.6 (0.7-3.5)	2.1 (0.9-4.2)	1.7 (1.0-3.0)	1.0 (0.5-1.9)	0.9 (0.5-1.7)	4.5 (1.9-8.0)	1.0 (0.5-1.0)	1.0 (0.9-1.2)
Hospitalizations												
Annualized event rate^b^	0.3 ± 4.5	0.3 ± 3.8	0.2 ± 2.6	0.2 ± 3.2	0.3 ± 3.0	0.2 ± 3.4	0.2 ± 3.2	0.2 ± 2.3	0.3 ± 1.8	0.28 ± 4.4	0.5 ± 3.0	0.5 ± 1.7
LOS per visit among patients with ≥ 1 visit, d	9.5 ± 16.9	9.9 ± 20.1	8.9 ± 16.1	8.8 ± 15.3	9.6 ± 16.6	9.9 ± 19.4	9.1 ± 16.5	9.2 ± 17.1	9.5 ± 15.5	9.5 ± 17.7	8.2 ± 18.4	9.0 ± 13.5
	6.0 (3.5-10.0)	6.0 (3.0-10.0)	5.5 (3.0-9.0)	5.5 (3.0-9.0)	6.0 (3.8-10.0)	6.0 (3.0-10.0)	5.5 (3.0-9.5)	5.7 (3.0-9.0)	6.0 (3.7-10.0)	6.0 (3.3-10.0)	5.0 (3.0-8.3)	5.8 (3.5-9.6)
ED visits												
Annualized event rate^b^	0.3 ± 3.9	0.2 ± 2.8	0.2 ± 2.5	0.3 ± 3.8	0.5 ± 3.0	0.2 ± 2.5	0.2 ± 2.9	0.3 ± 1.9	0.4 ± 4.2	0.3 ± 3.8	1.0 ± 4.3	1.0 ± 2.8
LOS per visit among patients with ≥ 1 visit, h	5.3 ± 3.7	5.2 ± 4.1	5.2 ± 4.0	5.3 ± 3.9	5.4 ± 3.5	5.4 ± 4.0	5.2 ± 3.9	5.2 ± 3.8	5.3 ± 3.6	5.3 ± 3.8	4.9 ± 3.8	5.1 ± 3.5
	4.7 (2.7-6.9)	4.4 (2.4-6.9)	4.4 (2.4-6.9)	4.7 (2.6-7.0)	4.8 (2.8-7.0)	4.6 (2.5-7.0)	4.5 (2.5-6.9)	4.5 (2.5-6.8)	4.7 (2.7-7.1)	4.6 (2.6-7.0)	4.2 (2.3-6.5)	4.5 (2.6-6.8)
Same-day surgery visits												
Annualized event rate b	0.0 ± 0.0	0.0 ± 0.0	0.0 ± 0.1	0.0 ± 0.0	0.0 ± 0.1	0.0 ± 0.1	0.0 ± 0.0	0.0 ± 0.1	0.0 ± 0.0	0.0 ± 0.1	0.0 ± 0.0	0.0 ± 0.1
General practitioner visits												
Annualized visit rate	2.3 ± 13.6	1.9 ± 12.7	1.8 ± 8.8	2.2 ± 10.2	3.2 ± 12.5	1.6 ± 10.7	1.8 ± 10.3	2.0 ± 9.9	2.8 ± 12.0	2.2 ± 13.5	4.4 ± 15.0	5.1 ± 14.8
Specialist visits												
Annualized visit rate	0.9 ± 8.9	0.7 ± 8.4	0.6 ± 4.8	0.8 ± 5.9	1.3 ± 7.4	0.6 ± 6.8	0.7 ± 6.4	0.7 ± 6.0	1.2 ± 7.4	0.8 ± 8.8	1.5 ± 7.5	2.4 ± 10.9

Data are presented as No., mean ± SD, or median (interquartile range). HCRU = health care resource use; LOS = length of stay; OCS = oral corticosteroid; SABA = short-acting beta-agonist.

a Medication history categories were defined using empirically derived thresholds and were based on the 1-y history.

b Total number of counts is based on one allowable encounter per provider or institution type, per patient per day.

c Patients can contribute to multiple strata; hence, their sum does not equal the All Patients column.

**Figure 2:**
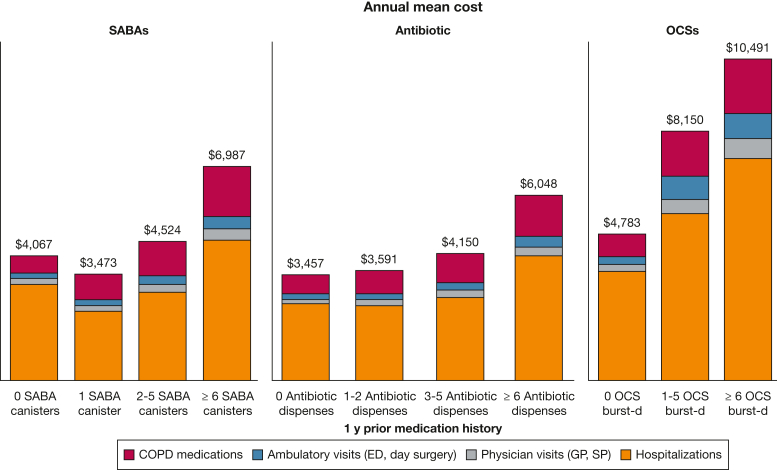
Bar graph showing annual mean COPD-specific health care costs per patient shown by medication dispensed in the past year for patients aged ≥ 35 years in Alberta, Canada, fiscal years 2011 through 2019. Mean costs are shown in Canadian dollars. GP = general practitioner; OCS = oral corticosteroid; SABA = short-acting beta-agonist; SP = specialist.

Similarly, annual COPD-specific HCRU costs related to medication history increased in a positively associated fashion. Costs were highest among patients with a history in any given year of ≥ 6 SABA dispensations (mean ± SD, $6,987 ± $34,685; median, $1,818 [IQR, $771-$4,073]), ≥ 6 antibiotic dispensations (mean ± SD, $6,048 ± $31,646; median, $1,055 [IQR, $236-$2,962]), or ≥ 6 OCS burst days (mean ± SD, $10,491 ± $31,627; median, $2,437 [IQR, $913-$7,656]). The lowest annual mean costs were among patients with a 1-year history of 1 SABA dispensation, 0 antibiotic dispensations, or 0 OCS burst days (Fig 2). Moving from lower to higher categories of prior 1-year SABA dispensation, an increasingly greater proportion of patients received dual- and triple-maintenance therapy (Table 4).

**Table 4:** Frequency of Any Subsequent COPD-Related Treatment Regimen Dispensed Given a 1-Year SABA Exposure History for Patients Aged ≥ 35 Years in Alberta, Canada, April 2011-March 2020^a^^,^^b^

Treatment During Follow-up	All Patients	1-Y History of SABA Use, No. of SABA Canisters			
		0	1	2-5	≥ 6
Total	188,969 (100.0)	158,327 (100.0)	87,608 (100.0)	83,526 (100.0)	45,659 (100.0)
Any monotherapy	75,250 (39.8)	54,509 (34.4)	33,007 (37.7)	29,164 (34.9)	12,152 (26.6)
ICS^c^	7,432 (3.9)	5,759 (3.6)	1,852 (2.1)	1,370 (1.6)	381 (0.8)
SABA or SAMA^d^	38,274 (20.3)	25,851 (16.3)	19,516 (22.3)	15,953 (19.1)	6,237 (13.7)
SABA	34,834 (18.4)	23,065 (14.6)	18,918 (21.6)	15,402 (18.4)	5,971 (13.1)
SAMA	4,233 (2.2)	3,249 (2.1)	738 (0.8)	656 (0.8)	289 (0.6)
LABA or LAMA^e^	39,932 (21.1)	27,784 (17.5)	14,671 (16.7)	14,627 (17.5)	6,714 (14.7)
LABA	1,889 (1.0)	1,322 (0.8)	648 (0.7)	598 (0.7)	255 (0.6)
LAMA	38,289 (20.3)	26,581 (16.8)	14,066 (16.1)	14,079 (16.9)	6,478 (14.2)
Any dual therapy	81,980 (43.4)	54,954 (34.7)	36,698 (41.9)	39,733 (47.6)	22,398 (49.1)
SABA plus SAMA	7,902 (4.2)	4,551 (2.9)	2,892 (3.3)	3,203 (3.8)	1,902 (4.2)
LABA plus LAMA	9,865 (5.2)	6,241 (3.9)	4,155 (4.7)	4,858 (5.8)	2,544 (5.6)
ICS plus SABA	12,486 (6.6)	6,997 (4.4)	6,593 (7.5)	6,934 (8.3)	3,438 (7.5)
ICS plus SAMA	819 (0.4)	588 (0.4)	190 (0.2)	151 (0.2)	86 (0.2)
ICS plus LABA	56,666 (30.0)	37,889 (23.9)	23,527 (26.9)	25,560 (30.6)	14,810 (32.4)
ICS plus LAMA	7,097 (3.8)	3,510 (2.2)	2,642 (3.0)	3,449 (4.1)	2,295 (5.0)
Any triple therapy	…	…	…	…	…
ICS plus LABA plus LAMA	63,166 (33.4)	39,119 (24.7)	29,839 (34.1)	37,754 (45.2)	26,844 (58.8)
Other therapy	…	…	…	…	…
ICS plus SABA plus SAMA	3,793 (2.0)	1,630 (1.0)	1,383 (1.6)	1,929 (2.3)	1,481 (3.2)

Data are presented as No. (%). ICS = inhaled corticosteroid; LABA = long-acting beta-2 agonist; LAMA = long-acting muscarinic antagonist; N/A = not applicable; SABA = short-acting beta-agonist; SAMA = short-acting muscarinic antagonist.

a Patients were classified as receiving a COPD regimen if treatment coverage spanned any part of the follow-up period.

b No. of patients across medication categories does not sum to total because patients may have contributed to multiple COPD regimens.

c By definition, ICS monotherapy could not occur concurrently with SABA, SAMA, LABA, or LAMA therapy; however, it could occur concurrently with maintenance medications, such as oral corticosteroids and antibiotics.

d By definition, SABA or SAMA monotherapy could not occur concurrently with LABA or LAMA therapy, or both.

e By definition, LABA or LAMA monotherapy could occur concurrently with SABA or SAMA therapy, or both.

## Discussion

This study described the burden of COPD using real-world data from Alberta, Canada, with a large population-based sample of > 180,000 patients. Nearly one-quarter of the patients met the COPD diagnosis criteria in hospital as opposed to physician visits, suggesting potential missed opportunities for earlier intervention to prevent serious AECOPDs. Overall, a positively associated relationship was demonstrated in which frequent dispensing of SABAs, antibiotics, and OCSs was associated with a greater burden of illness, with higher rates of severe AECOPDs, HCRU, and direct costs for patients with COPD. However, it should be noted that these are not causal effects because frequent dispensing of these medications are predictive only of patients at higher risk.

The associations between SABA (≥ 2 dispensations) or antibiotic (≥ 3 dispensations) history in a given year and severe AECOPDs within the next 90 days suggested a positively associated relationship independent of other risk factors examined, including previous severe AECOPD. The variable for previous exacerbations in our model was collinear with OCS burst days (because of the definition applied), and therefore attenuated the association between OCS burst day history and severe AECOPDs. Frequent dispensing of all three medications coincided with higher HCRU and costs, as expected given higher exacerbation rates. Associations between medication history and risk of COPD exacerbations have not been analyzed previously in the general population, but have been described in randomized controlled trials. Notably, the score to predict short-term risk of COPD exacerbations (SCOPEX) study focused on three multinational randomized controlled trials comprising 3,141 patients with moderate to very severe COPD, in whom higher use of mean daily SABA inhalations (in placebo) was associated independently with greater risks of exacerbation over 6 subsequent months.^15^

Based on the results of this retrospective study, SABA overuse may signal disease instability, particularly ≥ 6 SABA dispensations in a given year; even a threshold of 2 to 5 SABA dispensations demonstrated increased propensity for future severe AECOPDs. SABA use could be a valuable tool for health care professionals to identify patients at higher risk of future exacerbations, requiring further treatment and management optimization. In a study using administrative claims-based data in the United States, Sharafkhaneh et al^16^ found that previous SABA use of ≥ 1.5 doses/d was associated with a significantly high risk of exacerbations (mean ± SD annual rates of between 1.50 ± 1.45 and 2.55 ± 4.30 vs 1.35 ± 1.62 and 1.75 ± 2.88, respectively, with < 1.5 doses/d) and high annual COPD-related health care costs. In this study, one dose of metered-dose inhaler 90 SABA was defined as two puffs (one puff = 90 μg albuterol equivalent). Frequent SABA use as a reliever medication also was associated with short-term (≤ 21 days) and long-term (≤ 10 months) exacerbation risks in a study by Jenkins et al.^17^ The authors reported that even > 4 SABA inhalations/d were associated with high exacerbation risks, and risks increased with higher numbers of inhalations. In the study, patients were provided with albuterol metered-dose inhaler 90 μg × 2 inhalations to administer as reliever medication throughout the study. Our study findings provide a more practical and simple approach to SABA overuse as canister dispensation that is much easier for reviewing maintenance medication usage vs a calculation of daily SABA use that relies on patient recall that may be unreliable.

Frequent SABA use indicates that patients are seeking symptom relief, and this was demonstrated in the current study through higher proportions of patients receiving dual and triple therapy after more frequent SABA use, likely because of disease progression and increasing severity of symptoms. However, it should be noted that it is not SABA use itself that causes higher exacerbations; rather, SABA overreliance or misperception of using SABAs properly may be a signal of suboptimal disease management, the occurrence of mild to moderate exacerbations, and an opportunity to optimize the treatment regimen and patient education. A similar association was seen in the global SABA in Asthma (SABINA) study,^18^ which assessed SABA use as an indicator of asthma exacerbation risk. In that study, ≥ 3 SABA canisters were associated with an increased risk of severe asthma exacerbations, supporting our findings.

The positively associated relationship between antibiotic dispensations and exacerbation risk likely reflects periods with higher rates of prior low to moderate exacerbations. Although the risk-adjusted model controlled for prior rates of severe AECOPDs, moderate exacerbations, defined by the Canadian Thoracic Society guidelines as requiring the use of antibiotics or OCS,^9^ were not controlled for. It also suggests an ongoing unmet need among patients whose disease is uncontrolled, because exacerbation risk remained higher despite antibiotic use.

The association we observed between OCS burst days and exacerbation risk also is likely the result of prior moderate exacerbations for which OCSs are prescribed to manage. Although it is known that short-term use of OCSs plays an important role in the acute management of exacerbations,^19^ frequent past OCS dispensations resulting in ≥ 6 OCS burst days may be an indicator of disease progression or lack of symptom control, and thus future serious exacerbation events. Moreover, high dispensing of OCSs may increase the risk of adverse events, which also affects HCRU and costs.^20^

The study has several strengths. The first is the use of large, population-based administrative health care data sets with long follow-up. The province mandates reporting; therefore, missing values and loss to follow-up were not anticipated to impact the data quality. The Pharmaceutical Information Network database captured all pharmacy dispensations and detailed medication data, enabling robust analyses of real-world use of rescue medications. Instead of assigning a single exposure value across the entire follow-up, we identified COPD-related historical treatment in 90-day windows to consider time-varying risk and to capture treatment patterns better over time. Assessing the medication exposure preceding each follow-up period allowed us to identify the temporal associations between medication exposures and the risks of exacerbations and HCRU. Our analyses of OCS burst days not only incorporated dispensations, but also average daily dosages and durations of use. Further, a variety of patient and clinical characteristics were included in the risk-adjusted models, increasing the generalizability of these findings.

The study had limitations. Administrative data were collected for administrative purposes, which limited the type of information available. Medication dispensations may have overestimated the actual use. For example, errors in how pharmacists entered medication data may have occurred, and patient adherence is unknown. Further, we identified severe AECOPDs based on hospitalization and ED visits with COPD as the primary diagnosis, possibly underestimating exacerbation rates. Prior exacerbations have been demonstrated to be the biggest predictor of future exacerbations^21^; therefore, mild or moderate exacerbations that were uncontrolled for in the models likely drove the increased use of SABAs, antibiotics, and OCSs. Additionally, high rates of exacerbations have remained unreported, and in Canada, an estimated 68% of exacerbations were unreported by patients, causing significant harm.^22^^,^^23^ Moreover, it is difficult to determine if the patients were exposed to other chronic disease management, including vaccination, pulmonary rehabilitation, and so on. Finally, this study did not identify causal pathways between medication dispensation and exacerbations. Potential exists for residual confounding of unmeasured variables that are associated with both dispensing of COPD reliever medication and the study outcomes. For example, we did not measure patients’ reasons for frequent medication use, which are anticipated to have been key exposures to mild or moderate exacerbations. Future research should explore drivers for frequent dispensing in COPD and possible mediators of severe AECOPDs.

## Interpretation

Associations between COPD medications and severe AECOPDs and HCRU generally followed a positively associated trend. Specifically, a history of higher SABA (≥ 2 dispensations) or antibiotic (≥ 3 dispensations) use in a given year, or more OCS burst days (≥ 6), was associated with a greater burden of disease in terms of 90-day severe AECOPDs, HCRU, and costs. These real-world findings suggest that a history of frequently dispensed COPD rescue medications may be used as a tool or proxy for identifying a larger problem with disease control. Frequent use of SABAs, antibiotics, or OCSs may signal a need for improved management and treatment strategies. Clinicians and allied health care practitioners are encouraged to review the current maintenance treatment and management strategies for patients with a history of high dispensation of COPD rescue medications and to seek ways to decrease the potential for future serious exacerbations to improve patient outcomes.

## Funding/Support

This study was funded by AstraZeneca Canada.

## Financial/Nonfinancial Disclosures

The authors have reported to *CHEST Pulmonary* the following: P. M., D. F., A. G., E. G., and S. M. are employed by Medlior Health Outcomes Research Ltd., which received funding for the study from AstraZeneca Canada. M. B. received grants paid to his institution by CIHR and other pharmaceutical companies, including AZ, BI, GSK, Sanofi, and Mereo. I. M. received received grants paid to his institution by CIHR and other pharmaceutical companies, including BI, GSK, Sanofi, and Mereo M. B. volunteered at the Canadian Thoracic Society as an executive committee and board member unrelated to this work. A. R. and M. T. are employed by AstraZeneca Canada, who funded this study.

## Supplementary Data

e-Online Data
