# Supplementary material for: Short-Acting Beta-Agonists, Antibiotics, Oral Corticosteroids, and the Associated Burden of COPD
Source: CHEST Pulm. 2024 Feb 19;2(3):100042. doi: 10.1016/j.chpulm.2024.100042 (PMC13417807; doi:10.1016/j.chpulm.2024.100042)
Supplement: e-Online Data [file mmc1.docx]

**Supplementary Material 1: Datasets used for analysis**

Datasets provided by Alberta Health (Government of Alberta Ministry of Health) are described below.

| **Database** | **Description** |
| --- | --- |
| ABC Pharmacy Claims | Health benefit drug data for individuals with public coverage. |
| NACRS | Includes data from all emergency department visits and some outpatient-based ambulatory care, including information on services, diagnostic and procedure codes |
| DAD | Includes data from inpatient stays, including information on services, diagnostic and procedure intervention codes as well as length of stay |
| PIN | Includes information on medication dispenses and associated information at the pharmacy level (all private and public plans) |
| Population Registry | Includes basic demographic information, including age, gender, and zone |
| Practitioner Claims | Includes provider claims data for physicians and other providers for insured health services, and reports on provider and service data |
| Vital statistics – deaths | Includes death information |

Abbreviations: ABC: Alberta Blue Cross; DAD: Discharge Abstract Database; NACRS: National Ambulatory Care Reporting System; PIN: Pharmaceutical Information Network

**Supplementary Material 2: Methods for deriving the number of SABA canisters**

The number of SABA canisters was derived from administrative data as follows.

The Pharmaceutical Information Network (PIN) dataset and product monographs from Health Canada were used to identify the package size for each SABA drug identification number (DIN). The most common package size was 200 inhalations (doses). If the package size was not available, then a package size of 150 inhalations was assumed.

The (intermediate) quotient was then calculated as:

Quotient = (dispensed quantity ÷ package size)

The number of canister equivalents was calculated in a hierarchal fashion using the following algorithm:

1. If the quotient was a whole number, then it was assumed that the dispensed quantity was the number of inhalations.
2. If the quotient was not a whole number, and the unit was in canisters, then it was assumed that the dispensed quantity was the number of canisters.
3. If the quotient was not a whole number, and the unit was not in canisters, and the dispensed quantity was <30, and there were at least 7 days of supply for each canister, then it was assumed that the quantity was canisters.
4. Otherwise, it was assumed that the quantities were in doses, and the quotient of canister equivalents was assigned.

*Supplementary Table 2a: Examples of SABA canister calculations*

| Quotient  (dispensed quantity ÷ package size) | Quotient | Unit Type | Number of canisters |
| --- | --- | --- | --- |
| 600 ÷ 200 = 3 | whole number | not applicable | 3 |
| 2 ÷ 200 = 0.01 | not a whole number | canister | 2 |
| 20 ÷ 200 = 0.1 | not a whole number | not a canister | 20 |
| 250 ÷ 150 = 1.67 | not a whole number | not a canister | 1.67 |

**Supplementary Material 3: Methods for analyzing COPD-related medication regimens**

For the analysis of medication regimens, the first step was the identification of the start and end dates of each treatment medication type listed in the ***Supplementary Table 3a*** below. Treatment start was the first dispense date of a medication. Dispenses made on the same day for the same medication class with different Drug Identification Numbers (DIN) were assumed to be consumed concurrently. Treatment end was the last date of treatment recorded in the Pharmaceutical Information Network (PIN) dataset (dispense date plus treatment days). Gaps in treatment were allowed. Dual and triple lines of therapy were identified by anatomical therapeutic chemical (ATC) classification codes containing multiple medication types, or overlapping multiple medication dispenses of ≥14 days. A COPD regimen start was the date of first dispense or, for combination therapies, the date of first overlap. A COPD regimen end was the earlier of last day supply or start of a new regimen. If earlier prescribed medication was continued (the end date of the prescription overlapped with the new prescription by ≥1 day for OCS, or ≥14 days for other medication), then the new medication regimen included prior medication(s) as well as the new medication.

Treatment regimens were classified with the following rules: 1) to qualify as ICS monotherapy, ICS could not occur with SABA/SAMA/LABA/LAMA, but it could occur with OCS or antibiotics; 2) to qualify as SABA/SAMA monotherapy, LABA or LAMA could not be dispensed, and 3) when categories of LABA/LABA co-occurred with SABA/SAMA, then the medication regimen was classified as LABA/LABA only. The number and percentage of patients who had each treatment regimen was reported.

***Supplementary Table 3a*: *COPD-related medications***

| **Treatment Class** | **Medication** | **Anatomical therapeutic chemical (ATC) classification codes ^a^** |
| --- | --- | --- |
| P-4 Inhibitor | Phosphodiesterase inhibitors | C01CE |
| P-4 Inhibitor | Milrinone | C01CE02 |
| Oxygen Therapy | Oxygen | V03AN01 |
| Antibiotics | Antibiotics | J01 |
| SABA | Salbutamol | R03AC02 |
| SABA | Terbutaline | R03AC03 |
| SAMA | Ipratropium | R03BB01 |
| LAMA | Tiotropium | R03BB04 |
| LAMA | Aclidinium | R03BB05 |
| LAMA | Glycopyrronium | R03BB06 |
| LAMA | Umeclidinium | R03BB07 |
| LABA | Salmeterol | R03AC12 |
| LABA | Formoterol | R03AC13 |
| LABA | Indacaterol | R03AC18 |
| LABA | Olodaterol | R03AC19 |
| ICS | Beclometasone | R03BA01 |
| ICS | Budesonide | R03BA02 |
| ICS | Fluticasone | R03BA05 |
| ICS | Mometasone | R03BA07 |
| ICS | Ciclesonide | R03BA08 |
| ICS | Fluticasone furoate | R03BA09 |
| OCS | Fludrocortisone | H02AA02 |
| OCS | Betamethasone | H02AB01 |
| OCS | Dexamethasone | H02AB02 |
| OCS | Methylprednisolone | H02AB04 |
| OCS | Prednisolone | H02AB06 |
| OCS | Prednisone | H02AB07 |
| OCS | Triamcinolone | H02AB08 |
| OCS | Hydrocortisone | H02AB09 |
| OCS | Cortisone | H02AB10 |
| OCS | Methylprednisolone combinations | H02BX01 |
| SABA + SAMA | Salbutamol and ipratropium | R03AL02 |
| LAMA + LABA | Umeclidinium and vilanterol | R03AL03 |
| LAMA + LABA | Glycopyrronium and indacaterol | R03AL04 |
| LAMA + LABA | Aclidinium and formoterol | R03AL05 |
| LAMA + LABA | Tiotropium and olodaterol | R03AL06 |
| ICS + LABA | Salmeterol and fluticasone | R03AK06 |
| ICS + LABA | Formoterol and budesonide | R03AK07 |
| ICS + LABA | Formoterol and mometasone | R03AK09 |
| ICS + LABA | Vilanterol and fluticasone | R03AK10 |
| ICS + LABA + LAMA | Fluticasone furoate and Vilanterol and Umeclidinium bromide | R03AL08 |
| ICS + LABA + LAMA | Formoterol and budesonide and glycopyrronium bromide | R03AL11 |
| ICS + LABA + LAMA | Indacaterol, glycopyrronium bromide and mometasone | R03AL12 |

Abbreviations: SABA: short-acting beta agonist; SAMA: short-acting muscarinic antagonist; LAMA: long-acting muscarinic antagonist; LABA: long-acting beta agonist; ICS: inhaled corticosteroids.

^a^ The anatomical therapeutic chemical (ATC) classification codes in the table were selected based on the clinical opinions of co-authors. All Drug Identification Numbers mapping to respective ATC codes in the Pharmaceutical Information Network (PIN) dataset were included.

**Supplementary Material 4: Methods for estimating direct healthcare costs**

Costs associated with patients with COPD were estimated for inpatient hospitalizations, ED visits, practitioner claims, and medication dispenses. Hospital sector cost was estimated for each episode as the product of each patient’s Resource Intensity Weights (RIW) and the Alberta cost of a standard hospital stay in each fiscal year. COPD medication use was costed using DIN-level estimates derived from Alberta’s Blue Cross data for each fiscal year. Physician claims were costed at actual amount paid, or for records of shadow billing, standard system payment. Statistics Canada’s all-items Consumer Price Index was used to normalize all costs to April 1, 2022 constant Canadian dollars (Statistics Canada. Consumer Price Index (CPI) Statistics, Measures of Core Inflation and Other Related Statistics - Bank of Canada Definitions. 2021. Accessed April 6, 2021. <https://www150.statcan.gc.ca/t1/tbl1/en/tv.action?pid=1810025601>).

**Supplementary Material 5: Study flow diagram**


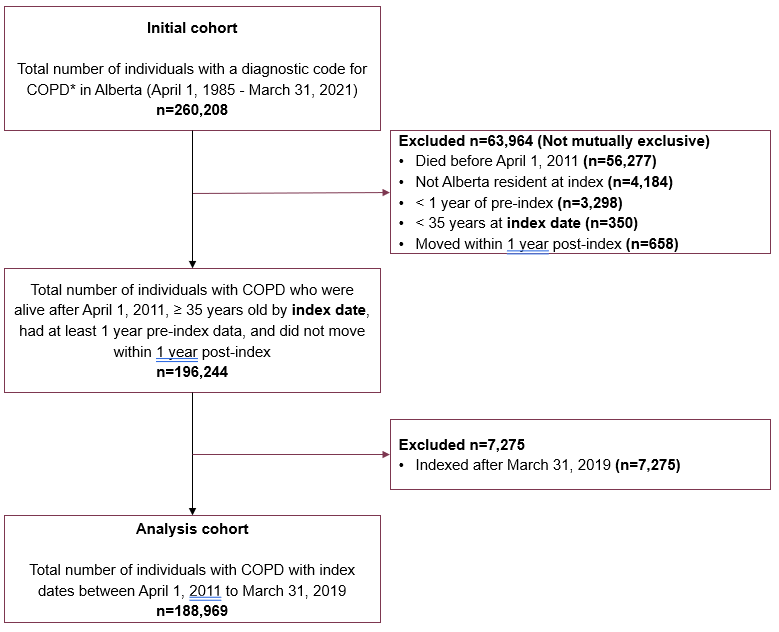


*Supplementary Figure 5a: Study flow diagram*

Abbreviations: COPD, chronic obstructive pulmonary disease

***** An International Classification of Diseases 10^th^ Revision (ICD-10-CA) diagnosis code for COPD (J41-J44) in any position in the discharge abstract database (DAD) where discharge date was the diagnosis date OR ≥ 2 ICD-9-CM diagnosis codes for COPD (491, 492, 496) in the primary position within a 2-year period in the Physician Claims dataset, where the second visit was the diagnosis date.

**Supplementary Material 6: Epidemiological methods and results**

Estimates of incident and prevalent cases of COPD were calculated from April 1, 2002, to March 31, 2020 among patients with COPD aged 35+ at diagnosis (or by April 1, 2022 for patients diagnosed earlier). Incidence and prevalence rates (per 100,000) were the number of cases (incident or prevalent) in a given fiscal year divided by the population of Alberta aged 35+ years from Statistics Canada. The annual incidence rate of COPD slightly increased after 2009 and then decreased after 2017 (range: 310 to 590 per 100,000). Annual prevalence rates of COPD generally increased (range: 4,532 to 5,489 per 100,000) over the study period.

**
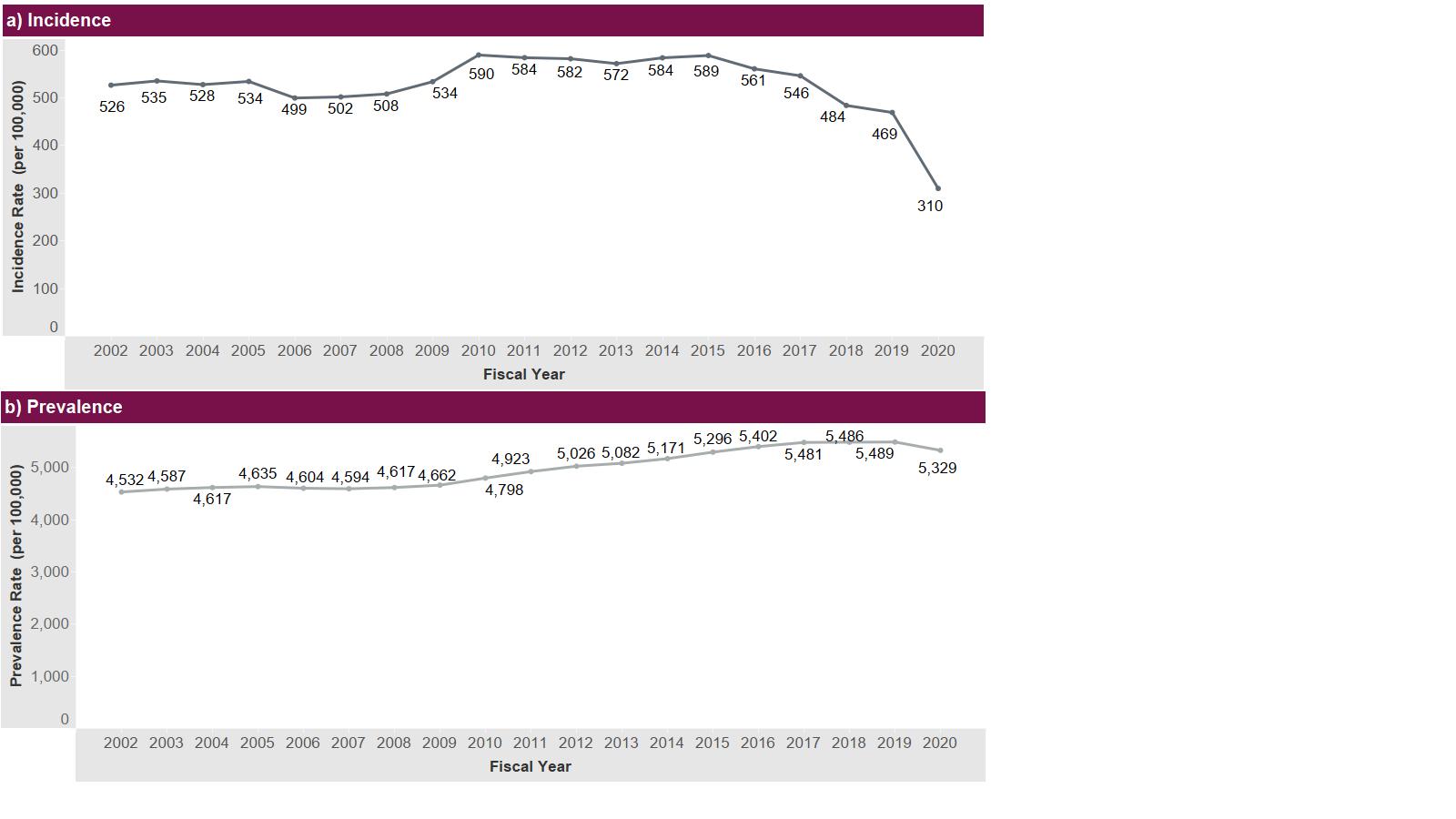
**

*Supplementary Figure 6a: Trends in COPD over time for individuals aged 35+ years in Alberta, Canada, fiscal years 2002-2020. Trends in incidence (panel a) and prevalence (panel b) are shown.*

**Supplementary Material 7: Additional CCI comorbidities**

| Characteristic | Overall  (n, %) |
| --- | --- |
|  |  |
| Total |  |
| Number of patients | 188,969 (100.0) |
| CCI comorbidities ^a^ |  |
| Hemiplegia or paraplegia | 750 (0.4) |
| Dementia | 3,383 (1.8) |
| Chronic pulmonary disease | 2,643 (1.4) |
| Rheumatic disease | 1,032 (0.5) |
| Peptic ulcer disease | 953 (0.5) |
| Renal disease | 3,027 (1.6) |
| Any malignancy, including leukemia and lymphoma | 5,271 (2.8) |
| Metastatic solid tumour | 1,682 (0.9) |
| Mild liver disease | 1,355 (0.7) |
| Moderate or severe liver disease | 462 (0.2) |
| AIDS/HIV | 83 (0.0) |

Abbreviations: AIDS/HIV: acquired immunodeficiency syndrome/human immunodeficiency virus; CCI: Charlson comorbidity index

^a^ Individuals were classified as having CCI comorbidities based on diagnoses recorded within 1 year before the index date based on hospital abstracts (DAD) only.

**Supplementary Material 8: Sensitivity analyses**

*Supplementary Table 8a: Sensitivity analysis estimating the associations between 90-day severe exacerbations and 1-year prior history of medication dispensation (excluding 1-year prior severe COPD exacerbation rate as a covariate) in patients with COPD in Alberta, Canada, April 2011 – March 2020 (n = 188,969)* *^a^*

|  | | **Risk-adjusted ^b^** | | |
| --- | --- | --- | --- | --- |
| **Medication dispensed (units)** | **1-year medication history** ^c^ | **IRR** | **95% LCL – UCL** | **P-value** |
| **SABA (canisters)** | 0 | 0.55 | 0.54 – 0.56 | <0.001 |
|  | 1 | 1.00 | Ref |  |
|  | 2-5 | 1.38 | 1.35 – 1.41 | <0.001 |
|  | 6+ | 2.22 | 2.17 – 2.28 | <0.001 |
| **Antibiotic (dispenses)** | 0 | 0.73 | 0.72 – 0.75 | <0.001 |
|  | 1-2 | 1.00 | Ref |  |
|  | 3-5 | 1.32 | 1.30 – 1.34 | <0.001 |
|  | 6+ | 1.72 | 1.68 – 1.76 | <0.001 |
| **OCS**  **(burst-days)** | 0 | 0.40 | 0.39 – 0.41 | <0.001 |
|  | 1-5 | 1.00 | Ref |  |
|  | 6+ | 1.32 | 1.28 – 1.36 | <0.001 |

Abbreviations: COPD: chronic obstructive pulmonary disease; IRR: incidence rate ratio; LCL: lower confidence limit; OCS: oral corticosteroids; Ref: reference group; SABA: short-acting beta agonist; UCL: upper confidence limit

^a^ Incidence rate ratios were modelled using a Poisson regression with severe exacerbations as the outcome variable.

^b^ Risk-adjusted model included: SABA dispenses (1-year history), antibiotic dispenses (1-year history), OCS burst-days (1-year history), sex, age in years at index date, calendar year of index date, and CCI at index.

^c^ Medication history categories were defined using empirically derived thresholds and were based on the 1-year history.

*Supplementary Table 8b: Sensitivity analysis estimating the associations between 90-day severe exacerbations and 1-year prior history of medication dispensation (including COPD maintenance treatment) in patients with COPD in Alberta, Canada, April 2011 – March 2020 (n = 188,969)* *^a^*

|  | | **Risk-adjusted ^b^** | | |
| --- | --- | --- | --- | --- |
| **Medication dispensed (units)** | **1-year medication history** ^c^ | **IRR** | **95% LCL – UCL** | **P-value** |
| **SABA (canisters)** | 0 | 0.67 | 0.66 | 0.69 |
|  | 1 | Ref | . | . |
|  | 2-5 | 1.26 | 1.24 | 1.29 |
|  | 6+ | 1.86 | 1.82 | 1.91 |
| **Antibiotic (dispenses)** | 0 | 0.76 | 0.75 | 0.77 |
|  | 1-2 | Ref | . | . |
|  | 3-5 | 1.27 | 1.25 | 1.29 |
|  | 6+ | 1.53 | 1.50 | 1.57 |
| **OCS**  **(burst-days)** | 0 | 0.48 | 0.47 | 0.49 |
|  | 1-5 | Ref | . | . |
|  | 6+ | 1.04 | 1.01 | 1.07 |

Abbreviations: COPD: chronic obstructive pulmonary disease; IRR: incidence rate ratio; LCL: lower confidence limit; OCS: oral corticosteroids; Ref: reference group; SABA: short-acting beta agonist; UCL: upper confidence limit

^a^ Incidence rate ratios were modelled using a Poisson regression with severe exacerbations as the outcome variable.

^b^ Risk-adjusted model included: SABA dispenses (1-year history), antibiotic dispenses (1-year history), OCS burst-days (1-year history), sex, age in years at index date, calendar year of index date, CCI at index, severe COPD exacerbation rate (1-year history), and dispensation of LABA and/or LAMA with or without ICS (1-year history).

^c^ Medication history categories were defined using empirically derived thresholds and were based on the 1-year history.

*Supplementary Table 8c: Sensitivity analysis estimating the associations between 90-day severe exacerbations and 1-year prior history of medication dispensation (including HCRU) in patients with COPD in Alberta, Canada, April 2011 – March 2020 (n = 188,969)* *^a^*

|  | | **Risk-adjusted ^b^** | | |
| --- | --- | --- | --- | --- |
| **Medication dispensed (units)** | **1-year medication history** ^c^ | **IRR** | **95% LCL – UCL** | **P-value** |
| **SABA (canisters)** | 0 | 0.59 | 0.58 | 0.61 |
|  | 1 | Ref | . | . |
|  | 2-5 | 1.35 | 1.33 | 1.38 |
|  | 6+ | 2.10 | 2.05 | 2.14 |
| **Antibiotic (dispenses)** | 0 | 0.85 | 0.84 | 0.87 |
|  | 1-2 | Ref | . | . |
|  | 3-5 | 1.22 | 1.20 | 1.24 |
|  | 6+ | 1.44 | 1.41 | 1.47 |
| **OCS**  **(burst-days)** | 0 | 0.54 | 0.52 | 0.55 |
|  | 1-5 | Ref | . | . |
|  | 6+ | 1.07 | 1.04 | 1.11 |

Abbreviations: COPD: chronic obstructive pulmonary disease; HCRU: healthcare resource utilization; IRR: incidence rate ratio; LCL: lower confidence limit; OCS: oral corticosteroids; Ref: reference group; SABA: short-acting beta agonist; UCL: upper confidence limit

^a^ Incidence rate ratios were modelled using a Poisson regression with severe exacerbations as the outcome variable.

^b^ Risk-adjusted model included: SABA dispenses (1-year history), antibiotic dispenses (1-year history), OCS burst-days (1-year history), sex, age in years at index date, calendar year of index date, CCI at index, severe COPD exacerbation rate (1-year history), any inpatient hospitalization (1-year history), any ED visit (1-year history) and any practitioner visit (1-year history).

^c^ Medication history categories were defined using empirically derived thresholds and were based on the 1-year history.
